# Trends in Bleeding Events Among Patients With Acute Coronary Syndrome in China, 2015 to 2019: Insights From the CCC-ACS Project

**DOI:** 10.3389/fcvm.2021.769165

**Published:** 2021-12-13

**Authors:** Xiao Wang, Guanqi Zhao, Mengge Zhou, Changsheng Ma, Junbo Ge, Yong Huo, Sidney C. Smith, Gregg C. Fonarow, Yongchen Hao, Jun Liu, Louise Morgan, Wei Gong, Yan Yan, Jing Liu, Dong Zhao, Yaling Han, Shaoping Nie

**Affiliations:** ^1^Center for Coronary Artery Disease, Beijing Anzhen Hospital, Capital Medical University, Beijing, China; ^2^Department of Epidemiology, Beijing Anzhen Hospital, Capital Medical University, Beijing Institute of Heart, Lung and Blood Vessel Diseases, Beijing, China; ^3^Department of Cardiology, Beijing Anzhen Hospital, Capital Medical University, Beijing, China; ^4^Zhongshan Hospital, Fudan University, Shanghai, China; ^5^Department of Cardiology, Peking University First Hospital, Beijing, China; ^6^Division of Cardiology, University of North Carolina, Chapel Hill, NC, United States; ^7^Divisions of Cardiology, University of California, Los Angeles, Los Angeles, CA, United States; ^8^International Quality Improvement Department, American Heart Association, New York, NY, United States; ^9^Cardiovascular Research Institute and Department of Cardiology, General Hospital of Northern Theater Command, Shenyang, China

**Keywords:** acute coronary syndrome, bleeding, temporal trend, antithrombotic therapy, outcome

## Abstract

**Objective:** Major bleeding is a common complication following treatment for an acute coronary syndrome (ACS) and is associated with increased mortality. We aimed to explore the temporal trend of bleeding events in relation to changes of therapeutic strategies among patients hospitalized for ACS in China.

**Methods:** The CCC-ACS project (Improving Care for Cardiovascular Disease in China–Acute Coronary Syndrome) is a collaborative initiative of the American Heart Association and the Chinese Society of Cardiology. We analyzed 113,567 ACS patients from 241 hospitals in China from 2015 to 2019. Major bleeding was defined as intracranial bleeding, retroperitoneal bleeding, a decline in hemoglobin levels ≥3 g/dL, transfusion with overt bleeding, bleeding requiring surgical intervention, and fatal bleeding. Kruskal–Wallis test was used to examine the trend of major bleeding over time.

**Results:** The rate of in-hospital major bleeding decreased from 6.3% in 2015 to 4.7% in 2019 (unadjusted OR = 0.74, 95% CI: 0.68–0.80, and *P* < 0.001). The relative changes were consistent across almost all subgroups including patients with NSTE-ACS and STEMI, although the trend was more pronounced in NSTE-ACS patients. The decrease in bleeding was accompanied by a decrease in use of GP IIb/IIIa inhibitors and parenteral anticoagulation therapy during hospitalization. The annual reduced risk of bleeding (OR = 0.91, 95% CI: 0.89–0.93) was attenuated after stepwise adjusting for baseline characteristics and antithrombotic treatments (OR = 0.95, 95% CI: 0.93–0.97), but did not change after adjusting for invasive treatment (OR = 0.95, 95% CI: 0.93–0.97).

**Conclusions:** There was a temporal reduction in in-hospital bleeding among Chinese ACS patients during the last 5 years, which was associated with more evidence-based use of antithrombotic therapies.

**Clinical Trial Registration:** https://www.clinicaltrials.gov, identifier: NCT02306616.

## Introduction

The use of intensive antithrombotic therapies coupled with revascularization has been shown to reduce ischemic risk after an acute coronary syndrome (ACS) (1), but such strategies are performed at the expense of an increased risk of bleeding (2). Recent analyses showed that major bleeding events were equivalent or even more prognostic for death than spontaneous myocardial infarction (3, 4).

In particular, East Asian people have different risk profiles for both thrombophilia and bleeding and might be more susceptible to bleeding (5). For potent antithrombotic therapy, various reports have failed to show a net clinical benefit in East Asian patients in contrast to similar studies in Caucasian patients (6). There are several studies describing temporal changes in bleeding rates among Western populations during the past decades (7–9), but no such data are available in East Asian countries. Therefore, we used data from the CCC-ACS project (Improving Care for Cardiovascular Disease in China–Acute Coronary Syndrome) (10) to delineate the temporal trend of in-hospital major bleeding in relation to development of therapeutic strategies and outcomes from 2015 to 2019 among Chinese patients hospitalized for ACS.

## Methods

### Study Design and Population

The CCC-ACS project (NCT02306616) is an ongoing nationwide registry and quality improvement project focusing on the quality of care for patients with ACS. The project was launched in November 2014 as a collaborative initiative of the American Heart Association and the Chinese Society of Cardiology. Details of the study design and methodology have been described elsewhere (10). In brief, the study included 159 tertiary hospitals and 82 secondary hospitals in China. In each hospital, the first 20 to 30 ACS patients of each month are consecutively recruited and identified based on principal discharge diagnosis. ACS was defined according to the guidelines published by the Chinese Society of Cardiology for the diagnosis and management of patients with ST-segment-elevation myocardial infarction (STEMI) and non-ST-segment elevation (NSTE)-ACS (11, 12). The Ethics Committee of Beijing Anzhen Hospital, Capital Medical University approved the study with a waiver for informed consent. The research was performed without patient or public involvement.

Overall, 113,650 inpatients with ACS from 241 hospitals were registered between November 2014 and December 2019. Of these, 113,567 patients were included in this study after excluding 83 (0.07%) patients with missing in-hospital outcomes.

### Data Collection

A standard web-based data collection platform (Oracle Clinical Remote Data Capture, Oracle) was used. Data elements included patients' characteristics, medical history, clinical presentation, diagnosis and risk evaluation, in-hospital management, discharge medications and in-hospital outcomes. Third-part clinical research associates performed regular quality audits to ensure that cases were reported consecutively rather than selectively.

### In-hospital Outcomes

Major bleeding was defined as a composite of intracranial bleeding, retroperitoneal bleeding, a decline in hemoglobin levels ≥3 g/dL during hospitalization, transfusion with overt bleeding, or bleeding requiring surgical intervention, and fatal bleeding. Major adverse cardiovascular events (MACE) included cardiac death, reinfarction, stent thrombosis, and ischemic stroke. Cardiac death was defined as death related to proximate cardiac causes, procedure-related complications, or any death unless an unequivocal non-cardiovascular cause could be established. All these outcomes were diagnosed by doctors during patients' hospitalization and recorded in medical records.

### Statistical Analysis

Changes in patients' characteristics, in-hospital management and outcomes were evaluated annually (from 2015 to 2019). The number of patients in 2014 was small and thus added to the number of patients in 2015. For in-hospital major bleeding, patients were also stratified by sex, age, type of ACS [STEMI, non ST segment elevation myocardial infarction (NSTEMI), unstable angina pectoris (UAP)], diabetes mellitus, hypertension, eGFR at admission, Killip class, percutaneous coronary intervention (PCI), type of P2Y_12_ inhibitor, parenteral anticoagulation therapy, and GPIIb/IIIa inhibitor use during hospitalization. Continuous variables were shown as mean [standard deviation (SD)] unless otherwise indicated. Categorical variables were presented as the number (percentage). Kruskal–Wallis test was used to examine the trend of major bleeding and ischemic outcomes over time.

To assess the effect of time on major bleeding, assuming a linear association for each 1-year time-block and outcome, a logistic regression model was fitted. These models explored the association for moving forward 1 year, with stepwise adjustments as follows: (1) crude; (2) age and gender; (3) baseline characteristics (diabetes mellitus, hypertension, eGFR <60 min per 1.73 m^2^, STEMI, and Killip class); (4) in-hospital antithrombotic treatments (GPIIb/IIIa inhibitor use, anticoagulant treatment, and ticagrelor use); and (5) invasive treatments [PCI (for STEMI, primary PCI), transradial access]. Standardization of rate of bleeding was performed using logistic regression models to account for the effect of differences in previously described patient characteristics and treatments throughout the observation period.

For variables with a missing rate of <15%, we imputed missing values using the sequential regression multiple imputation method implemented by IVEware software version 0.2 (Survey Research Center, University of Michigan, Ann Arbor, MI, USA). Missing rates of variables and strategies for the management of missing data are presented in Supplementary Table 1. Statistical analyses were performed using SAS 9.4 (SAS Institute, Cary, NC, USA), SPSS 26.0 (IBM SPSS Inc., Armonk, NY) and Stata 14.0 (Stata, College Station, TX, USA). Two-tailed *p*-values of < 0.05 were considered statistically significant.

## Results

Mean patient age of the whole cohort was 63.4 ± 12.4 years, and 27.1% were female. Most of the patients' characteristics showed only minor changes during the study period. There was a slight increase in the proportion of female patients and the prevalence of hypertension and previous PCI, and a decrease in the prevalence of previous stroke. The proportion of STEMI patients decreased steadily, associated with a temporal decline in patients with cardiac arrest (Table 1).

**Table 1:** Baseline characteristics.

	**2015 (*n* = 29,957)**	**2016 (*n* = 25,028)**	**2017 (*n* = 18,979)**	**2018 (*n* = 19,595)**	**2019 (*n* = 20,008)**	**2015–2019 (*n* = 113,567)**
Age, y	62.8 ± 12.5	62.9 ± 12.4	63.5 ± 12.4	64.0 ± 12.4	64.4 ± 12.3	63.4 ± 12.4
Female	7,516 (25.1)	6,265 (25.0)	5,117 (27.0)	5,554 (28.3)	6,010 (30.0)	30,462 (27.1)
Hypertension	19,446 (64.9)	16,372 (65.4)	12,637 (66.6)	13,378 (68.3)	13,779 (68.9)	75,612 (67.2)
Diabetes	13,090 (43.7)	11,101 (44.4)	8,829 (46.5)	9,156 (46.7)	9,158 (45.8)	51,334 (45.2)
Hyperlipidemia	25,325 (84.5)	21,015 (84.0)	15,829 (83.4)	16,386 (83.6)	16,396 (82.0)	94,951 (84.4)
Previous MI	2,415 (8.1)	1,826 (7.3)	1,572 (8.3)	1,826 (9.3)	1,885 (8.4)	9,524 (8.4)
Previous PCI	2,267 (7.6)	1,922 (7.7)	1,591 (8.4)	1,762 (9.0)	2,112 (10.6)	9,654 (8.5)
Previous CABG	154 (0.5)	126 (0.5)	104 (0.6)	118 (0.6)	91 (0.5)	593 (0.5)
Previous atrial fibrillation	798 (2.7)	538 (2.2)	424 (2.2)	486 (2.5)	460 (2.3)	2,706 (2.4)
Previous heart failure	730 (2.4)	426 (1.7)	375 (2.0)	570 (2.9)	595 (3.0)	2,696 (2.4)
Previous stroke	3,017 (10.1)	2,314 (9.3)	1,666 (8.8)	1,446 (7.4)	1,570 (7.9)	10,013 (8.9)
Previous peripheral vascular disease	347 (1.2)	217 (0.9)	173 (0.9)	184 (0.9)	229 (1.1)	1,150 (1.0)
Systolic blood pressure, mmHg	129.6 ± 23.5	130.5 ± 23.5	131.1 ± 23.5	132.3 ± 23.5	133.2 ± 24.1	133.2 ± 24.1
Diastolic pressure, mmHg	77.7 ± 14.4	78.2 ± 14.4	78.5 ± 14.2	79.3 ± 14.6	79.9 ± 14.7	79.9 ± 14.7
Heart rate, beats per min	77.2 ± 16.2	77.6 ± 16.3	77.6 ± 16.1	77.9 ± 16.3	78.3 ± 16.7	78.3 ± 16.7
STEMI	19,365 (64.6)	15,429 (61.7)	10,910 (57.5)	10,476 (53.5)	9,390 (46.9)	65,570 (58.3)
Cardiogenic shock	1,257 (6.5)	974 (6.3)	675 (6.2)	599 (5.7)	601 (6.4)	4,106 (6.3)
Cardiac arrest	687 (2.3)	439 (1.8)	195 (1.0)	125 (0.6)	111 (0.6)	1,557 (1.4)
**Killip class**						
Class I	20,213 (67.5)	17,096 (68.3)	12,489 (65.8)	12,298 (62.8)	12,637 (63.2)	74,733 (65.8)
Class II–III	8,066 (26.9)	6,614 (26.4)	5,484 (28.9)	6,230 (31.8)	6,208 (31.0)	32,602 (28.7)
Class IV	1,678 (5.6)	1,318 (5.3)	1,006 (5.3)	1,067 (5.5)	1,163 (5.8)	6,232 (5.5)
Renal insufficiency (eGFR <60 min per 1.73 m^2^)	5,467 (18.3)	4,362 (17.4)	3,375 (17.8)	3,554 (18.1)	3,580 (17.9)	20,338 (18.1)
Hemoglobin, g/dL	13.6 ± 2.1	13.6 ± 2.0	13.6 ± 2.0	13.7 ± 2.1	13.6 ± 2.1	13.6 ± 2.1

*CABG, coronary artery bypass grafting; eGFR, estimated glomerular filtration rate; MI, myocardial infarction; PCI, percutaneous coronary intervention*.

The use of antithrombotic therapies changed over time, with a fall in the use of anticoagulation therapy (77.5–60.6%) during hospitalization among all ACS patients. There was a marked decrease in the use of GP IIb/IIIa inhibitors over time (34.1–19.6%). Use of PCI was high in all ACS patients with extremely high proportion of radial access throughout the whole study period. The proportion of primary PCI in STEMI patients also increased over time (Table 2).

**Table 2:** In-hospital management^*^.

	**2015 (*n* = 29,957)**	**2016 (*n* = 25,027)**	**2017 (*n* = 18,979)**	**2018 (*n* = 19,595)**	**2019 (*n* = 20,008)**	**2015–2019 (*n* = 113,567)**
**Antiplatelet therapy**						
None	828 (2.8)	743 (3.0)	636 (3.4)	813 (4.2)	929 (4.6)	3,949 (3.5)
Aspirin only	499 (1.7)	572 (2.3)	606 (3.2)	984 (5.0)	1,184 (5.9)	3,845 (3.4)
P2Y_12_ receptor inhibitor only	799 (2.7)	592 (2.4)	524 (2.8)	511 (2.6)	546 (2.7)	2,972 (2.6)
DAPT	27,831 (92.9)	23,121 (92.4)	17,213 (90.7)	17,287 (88.2)	17,349 (86.7)	102,801 (91.4)
Ticagrelor	3,863 (13.5)	4,788 (20.2)	4,549 (25.7)	6,408 (36.0)	7,857 (43.9)	27,465 (24.4)
Glycoprotein IIb/IIIa inhibitors^†^	10,217 (34.1)	7,171 (28.7)	4,520 (23.8)	4,375 (22.3)	3,925 (19.6)	30,208 (26.9)
Anticoagulation therapy^‡^	23,207 (77.5)	18,205 (72.7)	13,149 (69.3)	12,477 (63.7)	12,130 (60.6)	79,168 (70.4)
UFH^‡^	1,159 (3.9)	837 (3.3)	657 (3.5)	483 (2.5)	932 (4.7)	4,068 (3.6)
LMWH^‡^	21,651 (72.3)	16,831 (67.3)	12,109 (63.8)	11,647 (59.4)	11,164 (55.8)	73,402 (65.3)
UFH or LMWH^‡^	22,449 (74.9)	17,385 (69.5)	12,511 (65.9)	11,935 (60.9)	11,804 (59.0)	76,084 (67.0)
Fondaparinux^‡^	405 (1.4)	346 (1.4)	270 (1.4)	321 (1.6)	143 (0.7)	1,485 (1.3)
Others^‡^	438 (1.5)	512 (2.1)	407 (2.1)	253 (1.3)	208 (1.0)	1,818 (1.6)
Warfarin	186 (0.6)	168 (0.7)	142 (0.8)	170 (0.9)	126 (0.6)	792 (0.7)
β-blockers	16,625 (55.5)	13,591 (54.3)	10,822 (57.0)	11,455 (58.5)	10,978 (54.9)	63,471 (55.9)
ACEI or ARB	14,439 (48.2)	11,755 (47.0)	9,178 (48.4)	9,502 (48.5)	8,737 (43.7)	53,611 (47.2)
Statins	28,094 (93.8)	23,349 (93.3)	17,658 (93.0)	18,322 (93.5)	18,665 (93.3)	106,088 (93.4)
Coronary angiography	21,378 (71.4)	18,981 (75.8)	14,893 (78.5)	15,262 (77.9)	14,761 (73.8)	85,275 (75.1)
PCI	19,372 (64.7)	18,142 (72.5)	13,641 (71.9)	13,473 (68.8)	12,917 (64.6)	77,545 (69.0)
DES^§^	16,556 (85.5)	15,197 (83.8)	11,687 (85.7)	11,934 (88.6)	11,192 (86.7)	66,566 (85.8)
BMS^§^	126 (0.7)	180 (1.0)	90 (0.7)	39 (0.3)	91 (0.7)	526 (0.7)
PTCA^§^	2,594 (13.4)	2,575 (14.2)	1,768 (13.0)	1,476 (11.0)	1,570 (12.2)	9,983 (12.9)
Others^§^	96 (0.5)	190 (1.1)	96 (0.7)	24 (0.2)	64 (0.5)	470 (0.6)
Primary PCI in STEMI patients	10,165 (52.5)	8,537 (55.3)	6,150 (56.4)	6,337 (60.5)	6,048 (64.4)	37,237 (56.8)
Thrombolysis	769 (2.6)	447 (1.8)	607 (3.2)	837 (4.3)	738 (3.7)	3,398 (3.0)
Transradial access	19,792 (92.6)	18,002 (94.8)	14,383 (96.6)	14,668 (96.1)	14,222 (96.3)	81,067 (95.1)

* *The medication use is defined as each medication used within 24 h after first medical contact unless otherwise indicated*.

† *Defined as use of glycoprotein IIb/IIIa inhibitors at any time during hospitalization*.

‡ *Defined as use of anticoagulant during hospitalization but not during index procedure*.

§ *Denominator is the total number of PCI patients enrolled in each year*.

*ACEI, angiotensin converting enzyme inhibitors; ARB, angiotensin receptor blockers; BMS, bare metal stent; DAPT, dual antiplatelet therapy; DES, drug eluting stent; LMWH, low molecular weight heparin; PCI, percutaneous coronary intervention; PTCA, percutaneous transluminal coronary angioplasty; STEMI, ST-segment elevation myocardial infarction; UFH, unfractionated heparin*.

### In-hospital Bleeding

**Figure 1:**
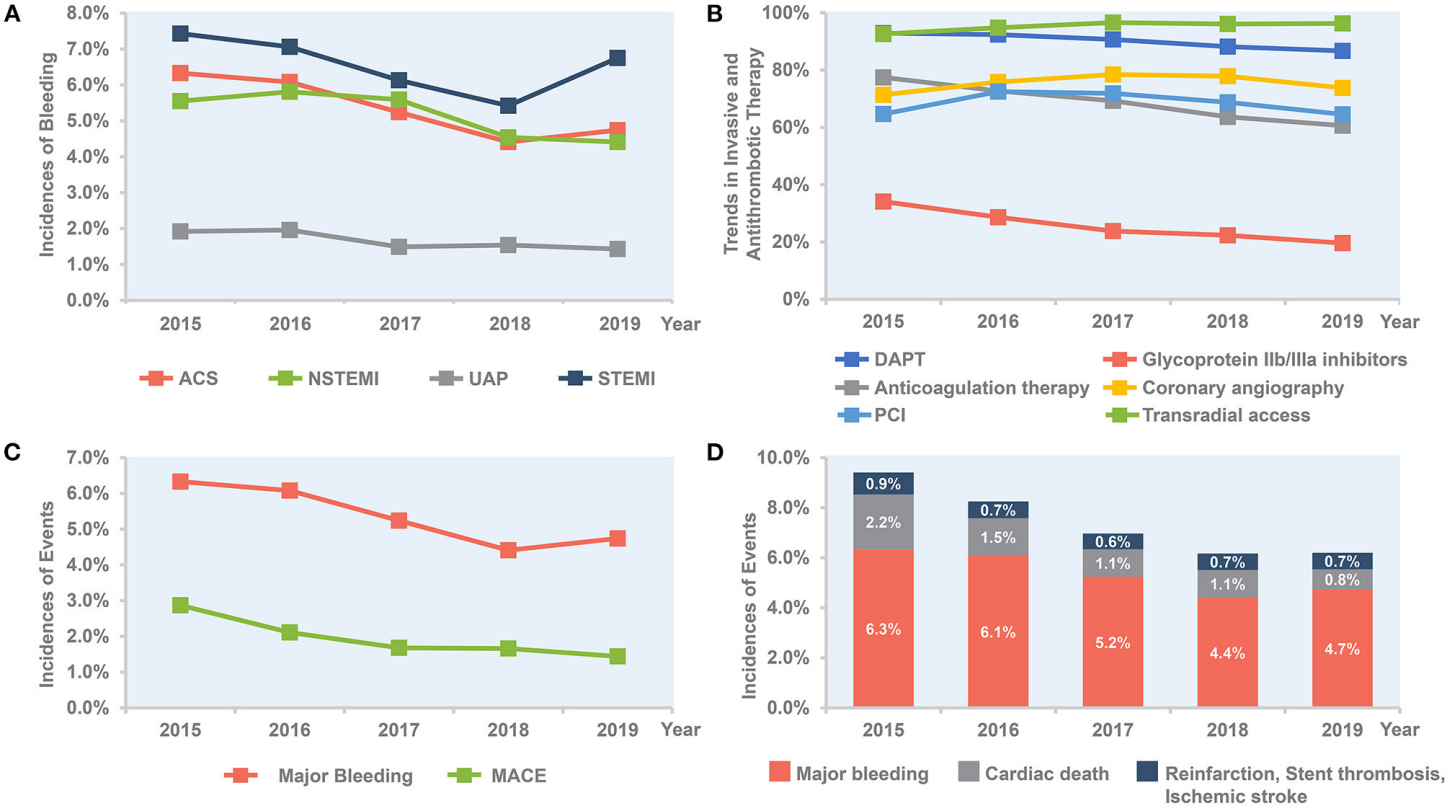
Temporal trend of in-hospital outcomes and treatment, 2015–2019. **(A)** Trends in rate of bleeding for all patients and per subgroup of STEMI, NSTEMI, and UAP. **(B)** Trends in invasive and antithrombotic therapy in all ACS patients. **(C)** Bleeding vs. MACE in all ACS patients. **(D)** Combined bleeding and ischemic outcomes in all ACS patients. ACS, acute coronary syndrome; DAPT, dual antiplatelet therapy; MACE, major adverse cardiovascular events; NSTE-ACS, non-ST-segment elevation acute coronary syndrome; NSTEMI, non-ST-segment elevation myocardial infarction; PCI, percutaneous coronary intervention; STEMI, ST-segment elevation myocardial infarction; UAP, unstable angina pectoris.

Overall, the rate of in-hospital major bleeding decreased from 6.3% in 2015 to 4.7% in 2019 (absolute change 1.6%, relative change 25.4%, unadjusted OR = 0.74, 95% CI: 0.68–0.80, and *P* < 0.001; Tables 3, 4; Figure 1A). A similar trend was found in patients with overt bleeding and those without overt bleeding but with decline in hemoglobin levels ≥3 g/dL (Table 4). The decrease of in-hospital bleeding occurred in parallel to the decreased use of GP IIb/IIIa inhibitors and anticoagulation therapy during hospitalization (Table 2; Figure 1B). Patients with NSTE-ACS (including NSTEMI and UAP) had a lower rate of bleeding than patients with STEMI, and similar trends were found in patients with NSTEMI and UAP (Table 4; Figure 1A). The trend of bleeding was consistent across almost all subgroups including age, sex, eGFR, P2Y12 receptor inhibitor, and anticoagulation therapy (Table 4).

**Table 3:** In-hospital outcomes in all ACS patients.

	**2015 (*n* = 29,957)**	**2016 (*n* = 25,027)**	**2017 (*n* = 18,979)**	**2018 (*n* = 19,595)**	**2019 (*n* = 20,008)**	***P* for trend**
Major bleeding	1,896 (6.3)	1,521 (6.1)	994 (5.2)	865 (4.4)	948 (4.7)	<0.001
MACE	859 (2.9)	528 (2.1)	318 (1.7)	326 (1.7)	288 (1.4)	<0.001
Cardiac death	650 (2.2)	383 (1.5)	212 (1.1)	217 (1.1)	167 (0.8)	<0.001
Reinfarction	124 (0.4)	75 (0.3)	72 (0.4)	86 (0.4)	79 (0.4)	0.14
Stent thrombosis	65 (0.2)	43 (0.2)	15 (0.1)	18 (0.1)	15 (0.1)	<0.0001
Ischemic stroke	87 (0.3)	60 (0.2)	39 (0.2)	32 (0.2)	49 (0.2)	0.06
All-cause death	689 (2.3)	412 (1.7)	249 (1.3)	272 (1.4)	278 (1.4)	<0.001

*ACS, acute coronary syndrome; MACE, major adverse cardiovascular events*.

**Table 4:** Trends in rate of bleeding by patients' characteristics and in-hospital management.

**Characteristics**	**Event rates**					**%Change from 2015 to 2019**	
	**2015** ** (*n* = 29,957)**	**2016** ** (*n* = 25,027)**	**2017** ** (*n* = 18,979)**	**2018** ** (*n* = 19,595)**	**2019** ** (*n* = 20,008)**	**Absolute Change**	**Relative Change**	***P* for trend**
Overall major bleeding	1,896 (6.3)	1,521 (6.1)	994 (5.2)	865 (4.4)	948 (4.7)	1.6	25.4	<0.001
Overt bleeding	718 (2.4)	560 (2.2)	310 (1.6)	287 (1.5)	335 (1.7)	0.7	29.2	<0.001
Non-overt bleeding with decline in hemoglobin levels ≥3 g/dL	1,178 (3.9)	961 (3.8)	684 (3.6)	684 (3.0)	613 (3.1)	0.8	20.5	<0.001
Sex								
Male	1,391 (6.2)	1,141 (6.1)	743 (5.4)	642 (4.6)	677 (4.8)	1.4	22.6	<0.001
Female	505 (6.7)	380 (6.1)	251 (4.9)	223 (4.0)	271 (4.5)	2.2	32.8	<0.001
Age								
≥75	458 (7.8)	380 (7.7)	263 (7.0)	222 (5.6)	250 (6.0)	1.8	23.1	<0.001
<75	1,438 (6.0)	1,141 (5.7)	731 (4.8)	643 (4.1)	698 (4.4)	1.6	26.7	<0.001
Type of ACS								
STEMI	1,439 (7.4)	1,089 (7.1)	669 (6.1)	568 (5.4)	634 (6.8)	0.6	8.1	<0.001
NSTE-ACS	457 (4.3)	432 (4.5)	325 (4.0)	297 (3.3)	314 (3.0)	1.3	30.2	<0.001
NSTEMI	388 (5.6)	368 (5.8)	279 (5.6)	237 (4.5)	240 (4.4)	1.1	20.5	<0.001
UAP	69 (1.9)	64 (2.0)	46 (1.5)	60 (1.5)	74 (1.4)	0.5	25.5	0.03
Diabetes mellitus								
Yes	1,001 (7.7)	773 (7.0)	532 (6.0)	443 (4.8)	468 (5.1)	2.6	33.8	<0.001
No	895 (5.3)	748 (5.4)	462 (4.6)	422 (4.0)	480 (4.4)	0.9	11.3	<0.001
Hypertension								
Yes	1,304 (6.7)	1,061 (6.5)	693 (5.5)	619 (4.6)	647 (4.7)	2.0	29.9	<0.001
No	592 (5.6)	460 (5.3)	301 (4.8)	246 (4.0)	301 (4.8)	0.8	14.3	<0.001
eGFR								
<60 mL/min per 1.73 m^2^	573 (10.5)	412 (9.5)	279 (8.3)	233 (6.6)	252 (7.0)	3.5	33.3	<0.001
≥60 mL/min per 1.73 m^2^	1,323 (5.4)	1,109 (5.4)	715 (4.6)	632 (3.9)	696 (4.2)	1.2	22.2	<0.001
Killip class								
I	1,071 (5.3)	890 (5.2)	574 (4.6)	491 (4.0)	515 (4.1)	1.2	22.6	<0.001
II–III	584 (7.2)	449 (6.8)	306 (5.6)	284 (4.6)	299 (4.8)	2.4	33.3	<0.001
IV	241 (14.4)	182 (13.8)	114 (11.3)	90 (8.4)	134 (11.5)	2.9	20.1	<0.001
PCI								
Yes	1,244 (6.4)	1,096 (6.0)	716 (5.3)	639 (4.7)	709 (5.5)	0.9	14.1	<0.001
No	652 (6.2)	425 (6.2)	278 (5.2)	226 (3.7)	239 (3.4)	2.8	45.2	<0.001
P2Y12 inhibitor								
Ticagrelor	321 (8.3)	345 (7.2)	258 (5.7)	273 (4.3)	392 (5.0)	3.3	39.8	<0.001
Clopidogrel	1,441 (5.8)	1,061 (5.6)	655 (5.0)	516 (4.5)	473 (4.7)	1.1	19.0	<0.001
Anticoagulation therapy								
Yes	1,511 (6.5)	1,167 (6.4)	737 (5.6)	593 (4.8)	613 (5.1)	1.4	21.5	<0.001
No	385 (5.7)	354 (5.2)	257 (4.4)	272 (3.8)	335 (4.3)	1.4	24.6	<0.001
UFH or LMWH								
Yes	1,464 (6.5)	1,121 (6.5)	709 (5.7)	570 (4.8)	602 (5.1)	1.4	21.5	<0.001
No	432 (5.8)	400 (5.2)	285 (4.4)	295 (3.9)	346 (4.2)	1.6	27.6	<0.001
GP IIb/IIIa inhibitor								
Yes	782 (7.7)	574 (8.0)	300 (6.6)	288 (6.6)	302 (7.7)	0.0	0.0	0.096
No	1,114 (5.6)	947 (5.3)	694 (4.8)	577 (3.8)	646 (4.0)	1.6	28.6	<0.001

*ACS, acute coronary syndrome; eGFR, estimated glomerular filtration rate; LMWH, low molecular weight heparin; NSTE-ACS, non-ST-segment elevation acute coronary syndrome; NSTEMI, non-ST-segment elevation myocardial infarction; PCI, percutaneous coronary intervention; STEMI, ST-segment elevation myocardial infarction; UAP, unstable angina pectoris; UFH, unfractionated heparin*.

**Figure 2:**
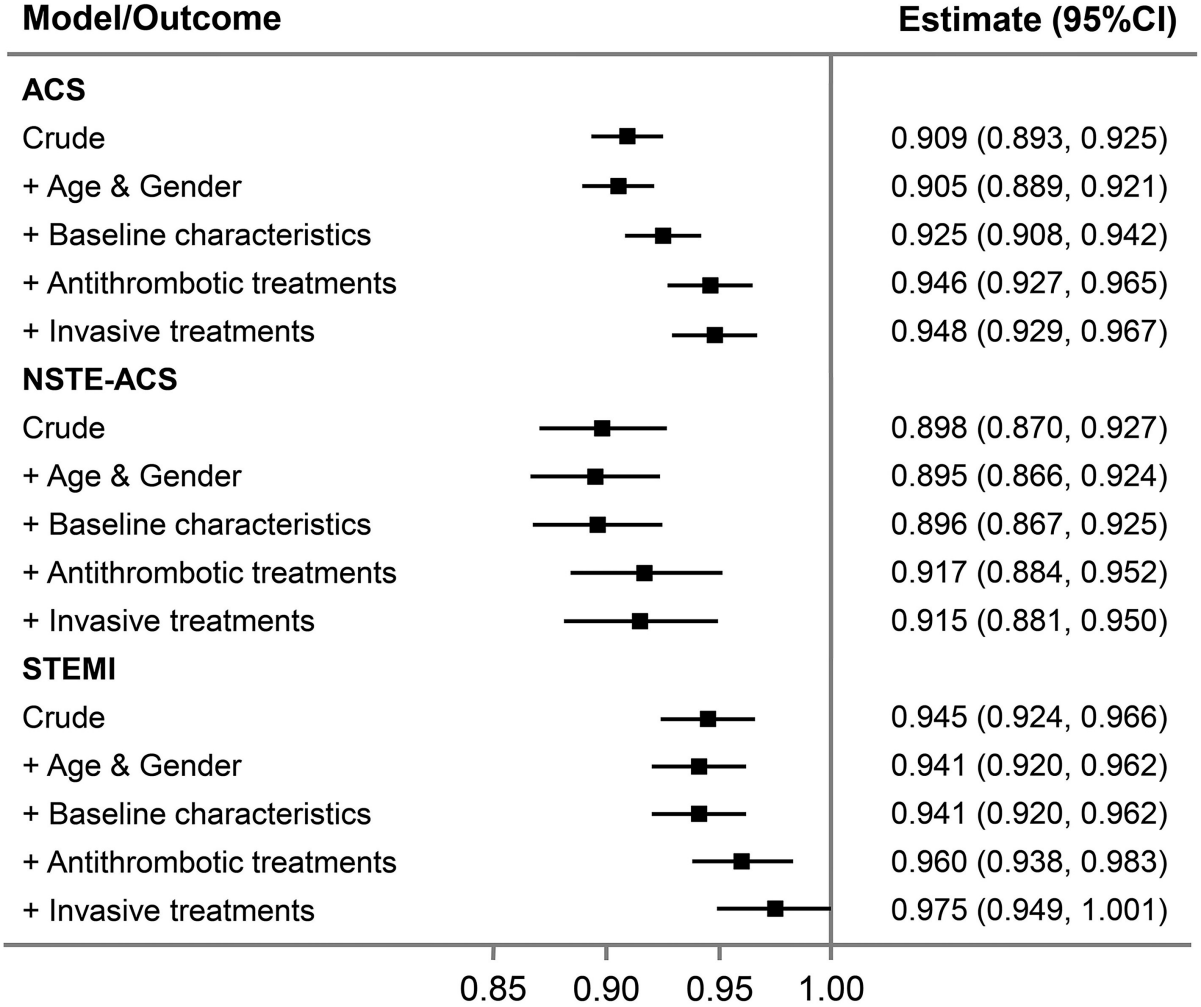
Association between 1 year change in time-period and major bleeding in all ACS patients and per subgroup of NSTE-ACS and STEMI. Stepwise adjustments as follows: (1) crude; (2) age and gender; (3) baseline characteristics (diabetes mellitus, hypertension, eGFR <60 min per 1.73 m^2^, STEMI, and Killip class); (4) in-hospital antithrombotic treatments (GPIIb/IIIa inhibitor use, anticoagulant treatment, and ticagrelor use); and (5) invasive treatments [PCI (for STEMI, primary PCI), transradial access]. ACS, acute coronary syndrome; NSTE-ACS, non-ST-segment elevation acute coronary syndrome; PCI, percutaneous coronary intervention; STEMI, ST-segment elevation myocardial infarction.

After adjustment for patient characteristics, medications, and interventions, the downward changes in bleeding were attenuated but remained for all ACS (Table 5). To evaluate the impact of changes in baseline characteristics and in-hospital management on outcomes, we did stepwise adjustments and analyzed the association between change in time-period and the risk of major bleeding. Each 1-year advancement was associated with reduced risk of bleeding in the crude analysis (OR = 0.91, 95% CI: 0.89–0.93) and after adjustment for changes in demographics (OR = 0.91, 95% CI: 0.89–0.92). The OR increased to 0.93 (95% CI: 0.91–0.94) after adjusting for baseline characteristics and further increased to 0.95 (95% CI: 0.93–0.97) after adjusting for antithrombotic treatments but did not change after adjusting for invasive treatment (OR = 0.95, 95% CI: 0.93–0.97; Figure 2). For patients with NSTE-ACS, only changes in antithrombotic therapy explained the time related reduction in bleeding. However, for patients with STEMI, the association was attenuated after adjusting for changes in antithrombotic therapy and was no longer significant after further adjusting for invasive treatments (mainly primary PCI; Figure 2).

**Table 5:** Standardized rate of bleeding in all ACS patients.

	**2015** ** (*n* = 29,957)**	**2016** ** (*n* = 25,027)**	**2017** ** (*n* = 18,979)**	**2018** ** (*n* = 19,595)**	**2019 ** **(*n* = 20,008)**
Model 1^*^	6.4 (6.1–6.7)	6.1 (5.8–6.4)	5.2 (4.9–5.6)	4.4 (4.1–4.7)	4.7 (4.4–5.0)
Model 2^†^	6.1 (5.9–6.4)	6.0 (5.7–6.3)	5.2 (4.9–5.6)	4.5 (4.2–4.8)	5.0 (4.7–5.3)
Model 3^‡^	5.8 (5.6–6.1)	6.0 (5.7–6.2)	5.4 (5.0–5.7)	4.6 (4.3–4.9)	5.2 (4.9–5.5)

* *Adjusted for age and gender*.

† *Adjusted for age, gender, and baseline characteristics (diabetes mellitus, hypertension eGFR <60 min per 1.73 m, STEMI, and Killip class)*.

‡ *Adjusted for age, gender, baseline characteristics, and in-hospital treatments (GPIIb/IIIa inhibitor use, anticoagulant treatment, ticagrelor use, PCI [for STEMI, primary PCI], and transradial access)*.

*ACS, acute coronary syndrome; eGFR, estimated glomerular filtration rate; PCI, percutaneous coronary intervention; STEMI, ST-segment elevation myocardial infarction*.

### Ischemic Outcomes

The annual rate of reinfarction, stent thrombosis, and ischemic stroke remains relatively low and constant. The incidence of MACE decreased steadily with an absolute decrease of 1.5% (from 2.9 to 1.4%), mainly driven by a decrease of cardiac death (from 2.2 to 0.8%; Table 3; Figure 1C). The rate of all-cause death also decreased over time (from 2.3 to 1.4%; Table 3). When combining ischemic and bleeding events together, a significant decrease in total events was also observed (Figure 1D).

## Discussion

The present analysis from the large CCC-ACS registry showed an absolute 1.6% decrease in major bleeding in relation to more evidence-based use of antithrombotic therapies in patients hospitalized for ACS in China. Simultaneously, the incidence of ischemic events including cardiac death decreased steadily during the study period. To the best of our knowledge, this is the first report to delineate the temporal changes of bleeding events in East Asian countries with a higher risk profile for bleeding.

The study shows a continuous reduction in bleeding from 2015 to 2019, which is accompanied by a decrease in use of GP IIb/IIIa inhibitors and parenteral anticoagulation therapy during hospitalization. These findings are in line with recent studies from the SWEDEHEART registry (7) and UK database (8) showing a decline in in-hospital bleeding associated with less use of GP IIb/IIIa inhibitors. Specifically, the use of GP IIb/IIIa inhibitors was still as high as 20% in 2019 compared with 4.7% in 2017/2018 in the SWEDEHEART registry (7). Currently, GP IIb/IIIa inhibitors are recommended on a “provisional” basis with decreased TIMI flow or new thrombus. Nevertheless, in many secondary hospitals in China, GP IIb/IIIa inhibitors were almost routinely used during and/or after PCI in STEMI or NSTEMI patients, which actually have no robust evidence but may increase bleeding. Furthermore, anticoagulation therapy (especially the use of LMWH) was regarded as a default therapy following ACS or PCI among many centers in China. However, this strategy was not associated with a lower risk of all-cause death or myocardial infarction but significantly increased risk of major bleeding in the era of PCI and DAPT (13). The decrease from 77.5 to 60.6% in anticoagulation therapy might also have driven the decline in bleeding events. These findings were supported by stepwise adjustments for antithrombotic therapies, which showed attenuated association of time-period and bleeding trend.

In contrast to prior reports (7, 8, 14), our study was performed on the background of high rates of radial access (95.1% from 2015 to 2019) with no significant change over the past 5 years. This strategy is intuitively a bleeding reduction strategy as recommended by guidelines (15, 16). Recent study of Japanese population also showed low risk of bleeding complications by use of transradial approach or vascular closure device (17). However, data on vascular closure device was not collected in the CCC-ACS registry. Due to high rate of radial access in our study, vascular access strategies were minimally associated with these observed reductions in bleeding rates over time. The use of new-generation DES featuring much lower strut thickness and biodegradable polymers or polymer- free designs allows the shortening of DAPT (18–20). Also, use of intravascular imaging to optimize the PCI procedure and achieve more complete endothelialization is important in patients at high risk of bleeding who might need a short duration of DAPT (21). Additional strategies include the use of proton pump inhibitors to reduce risk of gastrointestinal bleeding who need antithrombotic medications (22, 23). Although not collected in this project, these factors should be further addressed. Finally, there was a steep rise in the rate of bleeding in STEMI patients from 2018 to 2019. We noted that the proportion of cardiogenic shock in STEMI patients showed a decrease from 2015 to 2018 but a sharp rise from 2018 to 2019, which might at least partially explain this peculiar change. Other potential confounding factors may also take effects and should be further investigated.

In October 2016, the Chinese College of Cardiovascular Physicians Working Group on Thrombosis proposed a multidisciplinary expert consensus on the prevention and management of bleeding in patients with ACS receiving antithrombotic agents (24). The consensus proposed a multidisciplinary bleeding prevention and treatment framework with the cardiologist as the leader and several bleeding avoidance therapies including bleeding risk evaluation, tailored antithrombotic regimen, avoiding unnecessary in-hospital anticoagulation, crossover of anticoagulants, and use of GP IIb/IIIa inhibitors, and early use of PPI, which may partly explain the decline in bleeding rate after 2016.

The higher bleeding rate in our study compared with other reports maybe be partly attributed to higher proportion of STEMI patients and more use of GP IIb/IIIa inhibitors (≈20 vs. <5% in other registries) (7, 8) and anticoagulants. Actually, various studies have reported that East Asian people might be less prone to thrombosis and more prone to bleeding, especially in the context of potent antithrombotic therapy (25). In spite of this, our registry showed a faster and more substantial decrease in bleeding (2015–2019, from 6.3 to 4.7%, absolute change 1.6%) compared with the SWEDEHEART registry (2006/2007 to 2015/2016, from 2.0 to 1.3%, absolute change 0.7%).

On the other hand, the absolute reduction of MACE including cardiac death is also intriguing. In detail, the ischemic events including reinfarction, stent thrombosis, and ischemic stroke did not change a lot across the study. Although precisely causal link should be clearly established, we speculate the reduced rate of cardiac death might be in part owing to the decline of bleeding, as the latter may cause premature discontinuation of antithrombotic medications, reduced myocardial oxygen delivery due to hypotension and anemia, and blood transfusion (26). In the present study, we did not observe plateauing in bleeding rates, indicating that there is room for further lowering of event rates by further expansion of recommended therapies, which have not been fully exploited [e.g., high rate of GP IIb/IIIa inhibitors and anticoagulants use in 2019 compared to other registries (7, 8)].

### Limitations

First, bleeding events were self-reported and not adjudicated, possibly leading to underreporting. Second, the standardized bleeding definitions which were usually used in clinical trials were not available in our registry, although the definitions remained unchanged during the study period. However, our bleeding definition is similar to BARC 3 or 5 bleeding. Third, given the observational design of this study, the cause-effect relationship should not be established between change in antithrombotic therapy and temporal reduction in bleeding. Fourth, the information of IABP, which might be associated with bleeding, was only available from 2017 to 2019 and was not presented. Fifth, whether bleeding was access site related or not was not collected in this registry. Sixth, out of hospital events were also not reported. Seventh, data regarding the use of bivalirudin and new oral anticoagulants, which have been related to lower risk of bleeding, were not collected due to their infrequent use. Finally, the effects of timing, dosing, and crossover of anticoagulants and antiplatelets could not be assessed based on this registry.

## Conclusions

The present study shows a temporal reduction in in-hospital bleeding among Chinese ACS patients during the last 5 years. The changes in bleeding are largely associated with the decreased use of GP IIb/IIIa inhibitors and anticoagulation therapy. Given a higher risk profile for bleeding in the Chinese population, a more consistent and systematic application of evidence-based therapies and promotion of new treatment concepts are needed to further reduce bleeding risk and improve overall outcomes.

## Data Availability Statement

The datasets analyzed during the current study are not publicly available because of intellectual property rights, but are available from the corresponding author on reasonable request.

## Ethics Statement

The studies involving human participants were reviewed and approved by Ethics Committee of Beijing Anzhen Hospital, Capital Medical University. Written informed consent for participation was not required for this study in accordance with the national legislation and the institutional requirements.

## Author Contributions

XW, GZ, SN, and YaH: study concept and design. XW, GZ, MZ, YoH, JuL, JiL, DZ, SN, and YaH: acquisition, analysis, or interpretation of data. XW and GZ: drafting of the manuscript. XW, GZ, and SN had full access to all the data in the study and take responsibility for the integrity of the data and the accuracy of the data analysis. All authors critical revision of the manuscript for important intellectual content, read, and approved the final manuscript.

## Funding

This work was supported by a collaborative program of the American Heart Association (AHA) and the Chinese Society of Cardiology. The AHA was funded by Pfizer for the quality improvement initiative through an independent grant for learning and change.

## Conflict of Interest

GF consulted for Amgen, AstraZeneca, Bayer, Janssen, and Novartis and served on the AHA's Quality Oversight Committee. CM received honoraria from Bristol-Myers Squibb (BMS), Pfizer, Johnson & Johnson, Boehringer-Ingelheim (BI), Bayer and AstraZeneca for giving lectures. SN received research grants from the institution from Boston Scientific, Abbott, Jiangsu Hengrui Pharmaceuticals, China Resources Sanjiu Medical & Pharmaceuticals, East China Pharmaceuticals. The remaining authors declare that the research was conducted in the absence of any commercial or financial relationships that could be construed as a potential conflict of interest.

## Publisher's Note

All claims expressed in this article are solely those of the authors and do not necessarily represent those of their affiliated organizations, or those of the publisher, the editors and the reviewers. Any product that may be evaluated in this article, or claim that may be made by its manufacturer, is not guaranteed or endorsed by the publisher.
