# Supplementary material for: Trends in Bleeding Events Among Patients With Acute Coronary Syndrome in China, 2015 to 2019: Insights From the CCC-ACS Project
Source: Front Cardiovasc Med. 2021 Dec 13;8:769165. doi: 10.3389/fcvm.2021.769165 (PMC8710688; doi:10.3389/fcvm.2021.769165)
Supplement: Supplementary file 1 [file Table_1.DOCX]

**SUPPLEMENTAL MATERIALS**

**Supplemental Table 1. Missing rates of each variable and management of missing data (variables without any missing were not list in the following table)**

| **Variables** | **Missing, n (%)** | **Missing data pattern** | **Management of missing data** |
| --- | --- | --- | --- |
| Age | 429 (0.38) | MNAR (Missing not at random) | Patients with missing values were imputed using sequential regression multiple imputation method implemented by IVEware software |
| Systolic blood pressure | 190 (0.17) |  |  |
| Diastolic pressure | 223 (0.20) |  |  |
| Heart rate | 282 (0.25) |  |  |
| Hemoglobin | 4075 (3.59) |  |  |
| Cardiac arrest | 833 (0.73) |  |  |
| Renal insufficiency | 4629 (4.07) |  |  |
| Primary PCI | 1052 (2.00) |  |  |
| Aspirin | 867 (0.81) |  |  |
| Ticagrelor | 1604 (4.93) |  |  |
| Clopidogrel | 1060 (1.35) |  |  |
| Killip class | 14984 (13.18) | MAR (Missing at random) | Patients with acute heart failure were identified as Killip class 2-3 and patients with cardiogenic shock were identified as Killip class 4. If patients with unclear status of heart failure or cardiogenic shock, we imputed the missing values using sequential regression multiple imputation method implemented by IVEware software. Overall, 11.82% of data were derived from the heart failure and cardiogenic shock status and 1.37% of data were imputed. |
